# Health Beyond Disease: An Integrated Assessment of Quality of Life, Perceived Health, and Lifestyle Behaviours in a Peri-Urban Kenyan Population—A Pilot Study

**DOI:** 10.3390/ijerph23040438

**Published:** 2026-03-31

**Authors:** Emanuela Nuccio, Giovanni Boccia, Emanuela Santoro, Anna Esposito, Antonio Nigro, Vittoria Satriani, Roberta Manente, Angelo Cianciulli

**Affiliations:** 1 Department of Health Services, Medical Directorate of Hospital Facilities, A.O.R.N. “San Giuseppe Moscati”, 83100 Avellino, Italy; emanuelanuccio1986@gmail.com; 2 Department of Medicine, Surgery and Dentistry “Scuola Medica Salernitana”, University of Salerno, 84081 Salerno, Italy; gboccia@unisa.it (G.B.); vsatriani@unisa.it (V.S.); 3 San Giovanni di Dio e Ruggi d’Aragona University Hospital, 84081 Salerno, Italy; manente392@gmail.com; 4 Department of Translational Medical Sciences, University of Campania “Luigi Vanvitelli”, 80131 Napoli, Italy; espositoanna929@gmail.com; 5 Prevention Department, Complex Operational Unit (U.O.C.), Public Hygiene Local Health Authority, 84127 Salerno, Italy; at.nigro@aslsalerno.it

**Keywords:** quality of life, perceived health, cross-cultural health, WHOQOL-BREF, EQ-5D-5L, lifestyle behaviours, Kenya, public health

## Abstract

**Highlights:**

**Public health relevance—How does this work relate to a public health issue?**

- Kenya is experiencing an epidemiological transition with increasing non-communicable disease risk factors and lifestyle changes affecting population well-being.
- This study examines quality of life, perceived health status, and behavioral risk factors among adults attending a peri-urban primary care hospital, addressing a key community health context.

**Public health significance—Why is this work of significance to public health?**

- Evidence from sub-Saharan Africa often assesses quality of life, health status, or lifestyle separately; this study integrates all three dimensions using validated international instruments.
- The findings improve understanding of how behavioral and social determinants interact to influence health and well-being in low- and middle-income settings.

**Public health implications—What are the key implications or messages for practitioners, policy makers and/or researchers in public health?**

- Integrated screening of lifestyle risk factors and quality of life in primary care can support early identification of health needs in peri-urban African populations.
- Our results support the development of locally adapted prevention and health promotion strategies targeting behavioral risks and social determinants in Kenya.

**Abstract:**

**Background/Objective**: Health is a multidimensional construct shaped not only by clinical conditions but also by psychological, social, environmental and cultural factors. In low- and middle-income countries undergoing rapid epidemiological transition, understanding health requires integrated and culturally informed approaches. However, quality of life, perceived health status and lifestyle behaviors are often investigated separately, limiting the interpretation of well-being in specific local contexts. This study aimed to provide an integrated assessment of quality of life, perceived health status and lifestyle behaviours among adults attending a peri-urban public hospital in Kenya, using internationally validated instruments applied within a specific local cultural context. **Methods**: A cross-sectional observational study was conducted at Tigoni Level 4 Hospital, Kiambu County, Kenya. Adult outpatients (*N* = 40) were consecutively recruited. Quality of life was assessed using the WHOQOL-BREF, perceived health status using the EQ-5D-5L and EQ-VAS, and lifestyle behaviours using selected modules of the WHO STEPS instrument. Descriptive statistics were performed, and exploratory associations were examined using Spearman’s rank correlation coefficient. **Results**: Participants had a mean age of 35.9 ± 11.4 years, with a balanced gender distribution. Lifestyle risk factors were prevalent, including insufficient physical activity (40%) and overweight or obesity (>50%). WHOQOL-BREF scores revealed a heterogeneous profile, with relatively preserved social relationships and lower scores in the psychological and environmental domains. Pain/discomfort and anxiety/depression were the most frequently reported EQ-5D-5L problems. The mean EQ-VAS score was 68.2 ± 15.7. Perceived health was positively associated with physical and psychological quality of life, while higher body mass index was associated with lower physical quality of life. Mental health emerged as a cross-cutting factor across instruments. **Conclusions**: The findings highlight the multidimensional nature of health in a peri-urban Kenyan context and suggest the importance of considering local social and cultural influences when interpreting standardized health measures. Mental health and environmental conditions play a central role in shaping quality of life and perceived health, while lifestyle risk factors are already prevalent in a relatively young outpatient population. Integrating standardized health measures within a cross-cultural framework may support more holistic and person-centred approaches in primary care and public health in similar settings.

## 1. Introduction

Health is increasingly recognized as a multidimensional construct that extends beyond the mere absence of disease, encompassing physical, psychological, social, environmental, and cultural dimensions of well-being. The World Health Organization (WHO) defines health as “a state of complete physical, mental and social well-being and not merely the absence of disease or infirmity” [1].

Within this conceptual framework, indicators such as quality of life (QoL), perceived health status, and lifestyle behaviours have become essential for understanding population health, particularly in contexts characterized by social, economic, and epidemiological transitions. Low- and middle-income countries (LMICs), including Kenya, are currently facing a complex health scenario marked by the coexistence of infectious diseases and a rapidly increasing burden of non-communicable diseases (NCDs). This phenomenon, often described as the double burden of disease, is driven by urbanization, demographic changes, lifestyle transitions, and persistent structural inequalities [2,3]. In such settings, traditional biomedical indicators are insufficient to capture the full spectrum of health experiences, as they fail to account for psychosocial, behavioural, and environmental determinants that strongly influence individual and collective well-being.

A growing body of literature highlights that quality of life is not solely determined by clinical conditions but is profoundly shaped by social context, mental health, and access to resources. In Kenya and other sub-Saharan African countries, studies have shown that people living with chronic conditions such as HIV experience reduced quality of life due to stigma, economic insecurity, fear, and mental distress, even when clinical outcomes are well controlled [4,5]. This perspective has contributed to the emergence of the so-called “fourth 90”, emphasizing quality of life as a critical outcome alongside diagnosis, treatment, and viral suppression.

Mental health has consistently emerged as a key determinant of both perceived health and quality of life. High levels of depression, anxiety, and suicidal ideation have been reported among individuals seeking care from traditional healers and among internally displaced populations in Kenya, indicating a substantial burden of psychological distress that often remains underdiagnosed and undertreated [6,7]. These conditions not only affect emotional well-being but also influence functional ability, health-seeking behaviour, and adherence to preventive and therapeutic interventions. Environmental and social determinants are further intersected with health outcomes.

Limited access to basic services such as clean water, sanitation, transportation, and health care has been shown to negatively impact quality of life, particularly among women and socially vulnerable groups [8,9]. At the same time, lifestyle-related risk factors for NCDs—including physical inactivity, unhealthy diets, tobacco use, and alcohol consumption—are increasingly prevalent among young and middle-aged adults in sub-Saharan Africa [2,10,11].

These behaviours contribute not only to long-term morbidity but also to early declines in physical functioning, increased pain, and poorer self-rated health. Importantly, health-related quality of life (HRQoL) and perceived health are not universal constructs but are inherently shaped by cultural norms, social values, and shared meanings attributed to health, illness, and well-being. In peri-urban and multicultural settings such as those surrounding Nairobi, health perceptions are strongly influenced by collective resilience, family and community support, spiritual beliefs, and locally embedded coping strategies [12]. While standardized instruments such as the WHOQOL-BREF and EQ-5D-5L are internationally validated and designed for cross-cultural application, their interpretation is inevitably mediated by local cultural and contextual factors. Therefore, applying these tools in a Kenyan peri-urban context provides an opportunity not only to quantify health-related outcomes but also to explore how globally standardized measures capture locally grounded experiences of health and well-being.

Despite the growing availability of studies addressing quality of life, perceived health, or lifestyle behaviours in African contexts, most investigations examine these dimensions in isolation. There is a notable lack of integrated assessments that simultaneously consider quality of life, perceived health status, and lifestyle behaviours within the same population and health-care setting. This fragmentation limits the understanding of how behavioural, psychological, social, environmental, and cultural factors interact in shaping overall well-being and constrains the development of holistic and person-centred health strategies.

To address this gap, the present study adopts an integrated measurement approach using internationally validated instruments applied within a specific sociocultural context, combining the WHOQOL-BREF to assess quality of life, the EQ-5D-5L to capture perceived health status, and selected modules of the WHO STEPS instrument to evaluate lifestyle behaviours and NCD risk factors. Focusing on adults attending a peri-urban public hospital in Kenya, this study aims to provide a comprehensive snapshot of well-being and to explore the relationships between these dimensions. By situating standardized health measures within their local context, the study seeks to generate evidence relevant for strengthening primary care, prevention, and public health interventions in similar resource-constrained settings.

## 2. Materials and Methods

### 2.1. Study Design and Setting

A cross-sectional observational study was conducted to provide an integrated assessment of quality of life, perceived health status, and lifestyle behaviours among adult outpatients. The study was designed as an exploratory pilot investigation aimed at providing preliminary insights into the relationships between quality of life, perceived health status, and lifestyle behaviours in a real-world primary care context. Given the limited duration of data collection and the pragmatic nature of the field setting, a relatively small convenience sample was recruited. The study therefore primarily aims to generate hypotheses and inform the design of future larger-scale investigations. The study was carried out at Tigoni Level 4 Hospital, located in Kiambu County, north of Nairobi, Kenya. Level 4 hospitals represent an essential component of the Kenyan health system, offering general outpatient and inpatient services to heterogeneous populations from peri-urban and surrounding rural areas. The hospital catchment area reflects a context of ongoing epidemiological transition, characterized by the coexistence of communicable diseases, emerging non-communicable conditions, and marked socioeconomic diversity.

No formal sample size calculation was performed, as the study was designed as a pilot exploratory investigation aimed at generating preliminary data in a real-world clinical setting.

Given the exploratory nature of the research and the field-based data collection context, the study was designed as a pilot exploratory survey aimed at generating preliminary evidence on the relationships between quality of life, lifestyle behaviours, and perceived health in a peri-urban Kenyan outpatient population.

### 2.2. Data Collection Period

Data collection took place between 23 March and 2 April 2025, during routine outpatient department (OPD) activities. This period was selected to ensure feasibility and to minimize interference with standard clinical care.

### 2.3. Study Population and Eligibility Criteria

The study population consisted of adult patients attending the general outpatient department during the data collection period. Participants were recruited consecutively. Most visits to the outpatient department were related to routine medical consultations, follow-up of chronic conditions such as hypertension or diabetes, or general health assessments.

Inclusion criteria were as follows:

- age ≥ 18 years;
- ability to understand and respond to questionnaires in English or Swahili;
- provision of written informed consent.

Exclusion criteria included the following:

- acute or unstable clinical conditions that could compromise participation;
- cognitive impairment or severe illness preventing questionnaire completion;
- refusal to participate.

### 2.4. Data Collection Instruments

Three internationally validated instruments were used to capture complementary dimensions of health and well-being.

WHOQOL-BREF: Quality of life was assessed using the World Health Organization Quality of Life–BREF (WHOQOL-BREF), a 26-item questionnaire developed by the WHO for cross-cultural assessment of quality of life [13]. The instrument evaluates four domains—physical health, psychological well-being, social relationships, and environment—using a 5-point Likert scale. WHOQOL-BREF has demonstrated good reliability and validity across diverse cultural settings, including low- and middle-income countries (LMIC).

EQ-5D-5L: Perceived health status was measured using the EQ-5D-5L, a standardized instrument developed by the EuroQol Group to assess health-related quality of life across five dimensions: mobility, self-care, usual activities, pain/discomfort, and anxiety/depression [14]. Each dimension is rated across five levels of severity. Overall self-rated health was assessed using the EQ visual analogue scale (EQ-VAS), ranging from 0 to 100. The EQ-5D-5L has been widely used in international and cross-cultural research, including studies conducted in African and other low-resource settings. The EQ-5D-5L was administered using the descriptive system and EQ-VAS. As value sets vary substantially across countries and cultural contexts, and no Kenya-specific value set is currently available, utility index calculation was not performed [15].

Given the absence of a Kenya-specific EQ-5D-5L value set, analyses focused on descriptive profiles of health dimensions and EQ-VAS scores rather than the calculation of utility indices.

WHO STEPS Instrument: Lifestyle behaviours and non-communicable disease risk factors were assessed using selected modules of the WHO STEPwise approach to NCD risk factor surveillance (WHO STEPS) were used to assess key lifestyle-related risk factors. Specifically, the study included items derived from Module 1 (Tobacco use), Module 2 (Alcohol consumption), and Module 3 (Diet and physical activity) of the WHO STEPS instrument, version 2.1 [16]. These modules were used to collect information on smoking habits, alcohol consumption, dietary patterns, and physical activity levels. Specifically, modules assessing tobacco use, alcohol consumption, physical activity, and anthropometric measurements (height and weight for body mass index calculation) were included. The STEPS instrument is specifically designed for standardized, cross-cultural surveillance of behavioural risk factors in low- and middle-income countries and has been extensively applied in sub-Saharan African populations. Lifestyle behaviours were assessed using the WHO STEPwise approach to noncommunicable disease risk factor surveillance, a standardized and widely adopted framework for population-level assessment in low- and middle-income countries [17]. Physical activity was classified according to WHO recommendations. Participants reporting less than 150 min of moderate-intensity physical activity per week, or the equivalent combination of moderate and vigorous activity, were classified as having insufficient physical activity. Body mass index (BMI) was calculated as weight in kilograms divided by height in meters squared (kg/m^2^). BMI categories were defined according to WHO criteria: underweight (<18.5), normal weight (18.5–24.9), overweight (25.0–29.9), and obesity (≥30.0).

### 2.5. Data Collection Procedures

Data were collected through face-to-face, interviewer-administered questionnaires conducted by trained local healthcare professionals. Height and weight were measured during the visit using standard clinical equipment available in the outpatient department in order to calculate body mass index. Participants provided oral responses, which were recorded on standardized paper forms. Completed forms were subsequently entered into an electronic database for analysis.

To ensure data quality and consistency, interviewers received specific training on questionnaire administration, item interpretation, and scoring procedures. Data entry was cross-checked to minimize transcription errors.

Questionnaires were administered in either English or Swahili according to participant preference. Validated language versions of the WHOQOL-BREF and EQ-5D-5L instruments were used to ensure linguistic and conceptual equivalence across respondents.

### 2.6. Statistical Analysis

Descriptive statistics were used to summarize sociodemographic characteristics, lifestyle behaviours, quality of life, and perceived health status. Continuous variables were expressed as means and standard deviations (SD), while categorical variables were reported as frequencies and percentages.

WHOQOL-BREF domain scores were computed following WHO scoring recommendations. EQ-5D-5L results were presented as frequency distributions for each health dimension, and EQ-VAS scores were analysed as continuous variables.

Given the exploratory nature of the study and the limited sample size, non-parametric statistical methods were employed. Given the limited sample size, multivariable regression models were not implemented in order to avoid unstable parameter estimates and potential overfitting. Correlation analysis was therefore considered a more appropriate exploratory approach to examine relationships between health indicators. Spearman’s rank correlation coefficient was used to explore associations between quality of life domains, perceived health status, and selected lifestyle indicators. Statistical significance was set at *p* < 0.05. All statistical analyses were performed using R (version 4.3.2; R Foundation for Statistical Computing, Vienna, Austria) [18].

### 2.7. Ethics and Data Protection

This study consisted of a minimal-risk, non-interventional observational survey involving adult participants and the collection of fully anonymized data through internationally validated questionnaires. No clinical interventions were performed, no diagnostic or therapeutic procedures were introduced, and no identifiable personal information was collected.

Participation was entirely voluntary, and all participants were informed about the purpose and procedures of the study before data collection. Written informed consent was obtained from all participants prior to participation.

Given the anonymous nature of the survey, the absence of clinical interventions, and the minimal risk for participants, the study did not fall within the categories requiring formal ethics committee approval according to commonly applied institutional and international standards for anonymous observational survey research.

All data were collected anonymously and handled confidentially in accordance with the ethical principles of the Declaration of Helsinki and applicable data protection standards.

## 3. Results

### 3.1. Sample Characteristics and Lifestyle Profile

During the data collection period, 45 eligible patients were approached in the outpatient department. Of these, 40 agreed to participate and completed the survey, while five declined participation. No substantial missing data were observed for the variables included in the analysis. The mean age was 35.9 years (SD = 11.4), reflecting a relatively young outpatient population, although with a wide age range indicative of the heterogeneous catchment area of the hospital. Women accounted for 52.5% of the sample.

With regard to lifestyle behaviours, several modifiable risk factors emerged (Table 1). Approximately 22.5% of participants reported current tobacco use, while 35.0% reported current alcohol consumption. Notably, 40.0% of participants did not meet recommended levels of physical activity.

Body mass index distribution showed that more than half of the sample (50.0%) was overweight or obese, suggesting an emerging cardiometabolic risk profile even in this relatively young population. Overall, the lifestyle profile highlights the coexistence of behavioural risk factors typically associated with non-communicable diseases within a primary care outpatient setting.

### 3.2. Quality of Life Assessment (WHOQOL-BREF)

**Figure 1:**
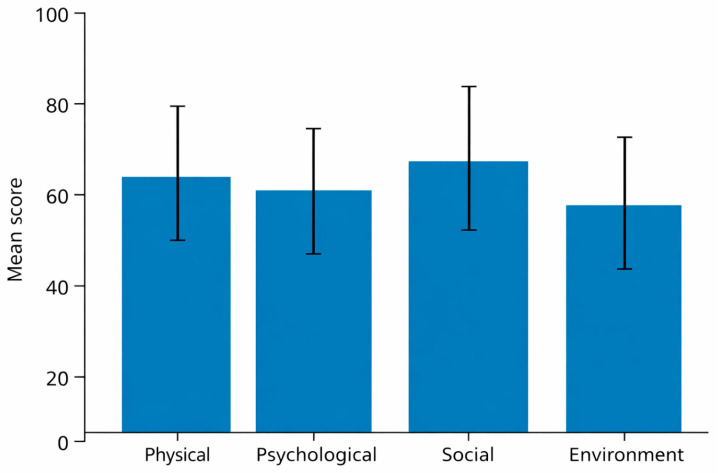
WHOQOL-BREF domain scores. Bar chart showing mean scores and standard deviations for physical, psychological, social relationships, and environmental domains.

WHOQOL-BREF domain scores revealed a heterogeneous quality of life profile across the four domains (Table 2; Figure 1).

The physical health domain showed moderate mean scores, with substantial variability among participants. Items related to energy levels, sleep quality, pain, and ability to perform daily activities contributed most to lower scores in this domain, particularly among individuals reporting sedentary behaviour or higher body mass index.

The psychological domain presented comparatively lower scores than the physical and social domains. Participants frequently reported negative feelings, reduced enjoyment of life, and difficulties with concentration, indicating a relevant burden of psychological distress within the sample.

In contrast, the social relationships domain emerged as relatively preserved. Most participants reported satisfactory personal relationships and perceived social support, suggesting that social cohesion and interpersonal networks may play a buffering role in perceived well-being.

The environmental domain displayed the lowest mean scores overall. Lower ratings were mainly driven by items related to financial resources, access to health services, transportation, safety, and living conditions. This finding underscores the impact of contextual and structural determinants on quality of life beyond individual health status.

### 3.3. Perceived Health Status (EQ-5D-5L)

**Figure 2:**
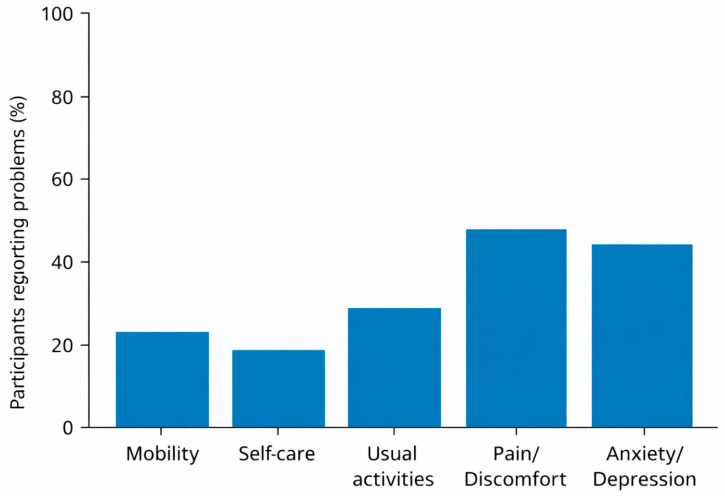
Prevalence of EQ-5D-5L-reported problems by dimension. Bar chart displaying the proportion of participants reporting at least one problem in each EQ-5D-5L dimension.

Analysis of the EQ-5D-5L dimensions showed that health problems were not evenly distributed across domains (Table 3; Figure 2).

The most frequently reported problems were related to pain/discomfort (37.5%) and anxiety/depression (35.0%), indicating that subjective suffering and emotional distress are common even among individuals attending outpatient services without acute clinical conditions.

Difficulties in mobility, self-care, and usual activities were less prevalent but still present in a non-negligible proportion of participants, suggesting early functional limitations in a subset of the population.

The mean EQ-VAS score was 68.2 (SD = 15.7), reflecting a moderate perception of overall health with wide inter-individual variability. Lower EQ-VAS scores were generally observed among participants reporting pain, emotional distress, and lower WHOQOL-BREF physical and psychological scores.

### 3.4. Relationships Between Quality of Life, Perceived Health, and Lifestyle Factors

**Figure 3:**
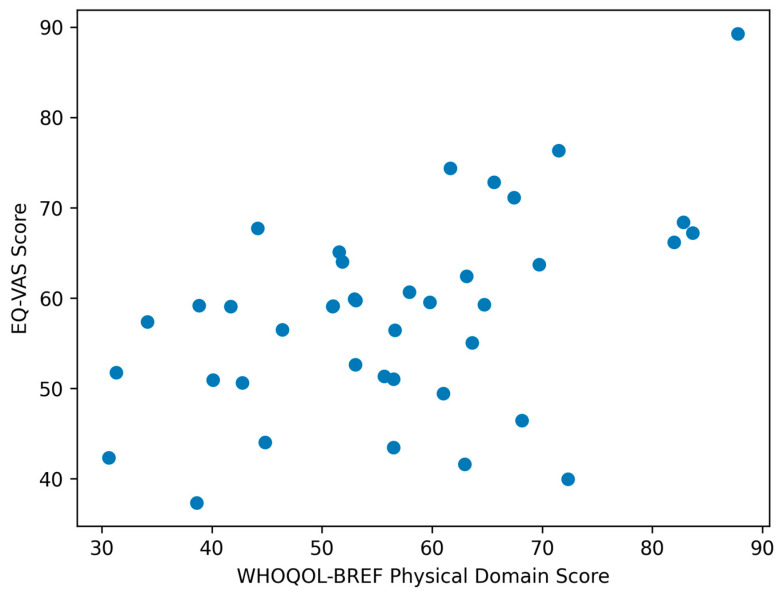
Scatter plot showing the relationship between EQ-VAS scores and the WHOQOL-BREF physical domain. Each point represents an individual participant. The plot illustrates the positive association observed in the correlation analysis.

Exploratory correlation analyses identified several patterns of association across measurement instruments, as reported in Table 4. Perceived overall health (EQ-VAS) showed a moderate to strong positive correlation with the physical domain of WHOQOL-BREF (ρ = 0.55, *p* < 0.01) and the psychological domain (ρ = 0.48, *p* < 0.01), indicating that better perceived health is closely aligned with both physical functioning and psychological well-being. To visually illustrate the relationship between perceived health and physical quality of life, a scatter plot was generated showing the association between EQ-VAS scores and the WHOQOL-BREF physical domain (Figure 3).

A negative association was observed between body mass index and the physical quality of life domain (ρ = −0.42, *p* < 0.05), suggesting that higher body weight is linked to poorer physical functioning and daily performance.

Participants reporting problems in the EQ-5D anxiety/depression dimension consistently showed lower WHOQOL-BREF psychological scores, reinforcing the internal coherence of the findings across instruments. No strong isolated associations were observed between single lifestyle behaviours (such as smoking or alcohol use) and quality of life domains; however, the overall pattern suggests that lifestyle-related risk factors may exert a cumulative rather than independent effect on perceived health and quality of life.

## 4. Discussion

This study provides an integrated and cross-cultural assessment of quality of life, perceived health status, and lifestyle behaviours among adult outpatients attending a peri-urban public hospital in Kenya. By combining WHOQOL-BREF, EQ-5D-5L, and WHO STEPS instruments, the findings offer a multidimensional perspective on health that goes beyond clinical indicators and highlights the interaction between behavioural, psychological, social, environmental, and cultural determinants.

### 4.1. Interpretation of Main Findings

The results reveal a heterogeneous quality of life profile, characterized by relatively preserved social relationships alongside lower psychological and environmental domain scores. This pattern is consistent with evidence from sub-Saharan African contexts, where social cohesion, family support, and community networks often play a central role in sustaining perceived well-being despite material constraints and psychosocial stressors. The relatively higher score observed in the social relationships domain may be interpreted, cautiously, as being consistent with the presence of supportive interpersonal and community networks described in previous literature from sub-Saharan African settings.

In contrast, the psychological domain emerged as one of the most vulnerable dimensions. Participants reported negative feelings, reduced enjoyment of life, and difficulties with concentration, findings that align with previous studies documenting a substantial burden of under-recognized mental health conditions in Kenya and similar settings. Importantly, previous research conducted in similar contexts has suggested that psychological distress may sometimes be described or perceived through somatic complaints or functional limitations rather than exclusively through biomedical or psychiatric categories. In this study, the observed pattern may be interpreted cautiously as being consistent with that literature, rather than as a directly measured cultural expression of distress.

The environmental domain yielded the lowest quality of life scores overall, underscoring the influence of structural and contextual determinants such as financial resources, access to services, transportation, safety, and living conditions. These findings reinforce the notion that individual health perceptions cannot be disentangled from broader social and environmental conditions, particularly in peri-urban areas experiencing rapid urbanization and uneven access to infrastructure. Similar patterns of compromised quality of life related to environmental and structural factors have been reported in African settings, where multimorbidity and social vulnerability significantly influence well-being across both rural and urban contexts [19].

### 4.2. Perceived Health Status and Functional Dimensions

Analysis of perceived health status using the EQ-5D-5L further supports the multidimensional nature of health in this population. Pain/discomfort and anxiety/depression were the most frequently reported problems, while limitations in mobility, self-care, and usual activities were less prevalent but still present. This distribution suggests that subjective suffering and emotional distress may precede or outweigh overt functional disability in relatively young outpatient populations.

The moderate mean EQ-VAS score, coupled with wide inter-individual variability, highlights differences in how individuals evaluate their overall health. The positive correlations between EQ-VAS scores and the physical and psychological domains of WHOQOL-BREF indicate internal coherence across instruments and suggest that perceived health is closely linked to both functional capacity and mental well-being.

### 4.3. Lifestyle Behaviours and Early Risk Patterns

Lifestyle-related risk factors were prevalent in the study sample, with a substantial proportion of participants reporting insufficient physical activity and overweight or obesity. Although no strong isolated associations were observed between individual behaviours and quality of life domains, the negative association between body mass index and physical quality of life suggests that cardiometabolic risk factors may already be exerting an impact on daily functioning and perceived health.

These findings are particularly relevant given the relatively young mean age of the sample and point to the early emergence of non-communicable disease risk profiles in peri-urban Kenyan populations. From a public health perspective, this underscores the importance of integrating lifestyle counselling, physical activity promotion, and preventive strategies into routine primary care services.

### 4.4. Cross-Cultural Considerations

A relevant aspect of this study lies in the application of internationally validated health measurement instruments within a specific sociocultural context. While WHOQOL-BREF, EQ-5D-5L, and WHO STEPS are internationally validated instruments, their interpretation is inevitably shaped by local cultural norms, values, and lived experiences. The coexistence of relatively preserved social well-being with lower psychological and environmental quality of life suggests that standardized measures may reflect health experiences that are shaped by local social and cultural contexts.

Rather than questioning the validity of these instruments, the findings support the need to interpret quantitative scores within the broader social, environmental, and cultural context in which health perceptions are formed. In this sense, the study supports the use of standardized tools as part of a culturally informed assessment framework, particularly when complemented by an understanding of social and environmental determinants. It should be noted that the present study does not aim to perform a formal cross-cultural comparison. Rather, it explores how internationally standardized health instruments capture health-related perceptions within a specific local setting.

### 4.5. Strengths and Limitations

First, the relatively small sample size (*N* = 40) reflects the exploratory and pilot nature of the study and limits the statistical power of the analyses. Therefore, the results should be interpreted cautiously and primarily as hypothesis-generating findings rather than definitive evidence.

This study has several strengths. First, it adopts an integrated measurement approach that simultaneously captures quality of life, perceived health status, and lifestyle behaviours. Second, it contributes empirical data from a peri-urban Kenyan setting, a context that remains underrepresented in the quality-of-life literature. Third, the use of internationally validated instruments enhances comparability with other studies.

However, several limitations should be acknowledged. First, the relatively small sample size (N = 40) limits statistical power and restricts the generalizability of the findings. The study should therefore be interpreted as an exploratory pilot investigation aimed at generating preliminary hypotheses rather than providing definitive population-level estimates. Second, participants were recruited using a consecutive convenience sampling strategy within a single outpatient setting, which may introduce selection bias and limit the representativeness of the sample. Third, the cross-sectional design precludes causal inference between lifestyle behaviours, perceived health status, and quality of life outcomes. Lifestyle behaviours were self-reported and may be subject to recall or social desirability bias. Additionally, the absence of a Kenya-specific EQ-5D-5L value set prevented the calculation of utility indices, restricting analyses to descriptive profiles and EQ-VAS scores.

Furthermore, the results reflect the characteristics of a single peri-urban outpatient population and should not be generalized to the wider Kenyan population or to other health-care settings without further investigation.

Future studies with larger and more representative samples will be necessary to confirm the observed associations and to improve the generalizability of the findings.

### 4.6. Public Health Implications and Future Directions

Despite these limitations, the findings have important implications for primary care and public health practice. Future studies with larger samples should consider multivariable analytical approaches, including multiple linear regression or structural modelling, to better explore the independent contribution of lifestyle behaviours, environmental conditions, and psychological factors to quality of life and perceived health outcomes. The prominent role of mental health and environmental factors suggests that interventions aimed at improving well-being should extend beyond biomedical care to include psychosocial support and attention to structural determinants. Integrating mental health screening and counselling into outpatient services may represent a feasible and impactful strategy in similar settings.

Future research should consider longitudinal designs to examine changes in quality of life and perceived health over time and to explore causal pathways between lifestyle behaviours and well-being. Larger multicentre studies could enhance generalizability and support the development of locally relevant benchmarks for quality-of-life assessment. Qualitative approaches may also complement quantitative findings by providing deeper insight into culturally specific meanings of health and resilience.

## 5. Conclusions

This exploratory study provides preliminary insights into the multidimensional nature of health in a peri-urban Kenyan outpatient population. By integrating quality of life, perceived health status, and lifestyle behaviours within a single analytical framework, the findings provide a nuanced picture of well-being that extends beyond traditional clinical indicators.

The results show that, in this peri-urban Kenyan outpatient population, psychological distress and environmental constraints represent the most fragile dimensions of quality of life, while social relationships remain relatively preserved. This pattern may be interpreted as reflecting the coexistence of vulnerability and potentially protective relational resources, as suggested in the previous literature, although such mechanisms were not directly measured in the present study. At the same time, the high prevalence of pain/discomfort and anxiety/depression highlights an often-under-recognized burden of subjective suffering that may not be fully captured by routine biomedical assessments.

Lifestyle-related risk factors, including insufficient physical activity and overweight or obesity, were already prevalent in a relatively young population, indicating the early emergence of non-communicable disease risk profiles. Although the cross-sectional nature of the study does not allow causal inference, the observed associations between body mass index, physical quality of life, and perceived health suggest that behavioural and metabolic factors may begin to influence daily functioning and well-being well before the onset of overt disease.

Importantly, this study demonstrates the value of applying standardized health-related quality of life instruments within a culturally informed framework. While tools such as the WHOQOL-BREF and EQ-5D-5L enable comparability across settings, their interpretation must be grounded in local social, environmental, and cultural contexts. The findings suggest that quantitative scores are more meaningfully interpreted when considered alongside the lived, social, and structural contexts that may shape health perceptions. From a public health and primary care perspective, these results support the need for more holistic and person-centred approaches to care. Interventions aimed at improving population health in similar peri-urban contexts should integrate mental health support, address environmental and social determinants, and promote healthy lifestyles as part of routine outpatient services. Such strategies may contribute not only to disease prevention but also to the enhancement of overall well-being and perceived health.

In conclusion, an integrated and cross-cultural assessment of health provides critical insights into the complex interplay between lifestyle behaviours, quality of life, and perceived health. These findings should be interpreted cautiously and primarily as hypothesis-generating observations within a single outpatient setting.

## Figures and Tables

**Table 1:** Sociodemographic and lifestyle characteristics of the sample (*N* = 40).

Variable	Value
Age, mean ± SD (years)	35.9 ± 11.4
Female sex, *N* (%)	21 (52.5)
Current smokers, n (%)	9 (22.5)
Current alcohol consumers, n (%)	14 (35.0)
Insufficient physical activity, n (%)	16 (40.0)
Body Mass Index (BMI) category	*N* (%)
Normal weight	18 (45.0)
Overweight	12 (30.0)
Obesity	8 (20.0)
Underweight	2 (5.0)

Note: Values are expressed as mean ± standard deviation for continuous variables and number (percentage) for categorical variables.

**Table 2:** WHOQOL-BREF domain scores (mean ± SD).

Domain	Mean ± SD
Physical health	61.4 ± 14.2
Psychological	58.1 ± 13.6
Social relationships	65.7 ± 15.1
Environment	54.3 ± 12.8

Note: Higher scores indicate better perceived quality of life.

**Table 3:** Distribution of EQ-5D-5L-reported problems by dimension.

EQ-5D-5L Dimension	Participants Reporting ≥ 1 Problem, n (%)
Mobility	6 (15.0)
Self-care	4 (10.0)
Usual activities	7 (17.5)
Pain/discomfort	15 (37.5)
Anxiety/depression	14 (35.0)

EQ-VAS score (mean ± SD): 68.2 ± 15.7.

**Table 4:** Exploratory correlations between quality of life, perceived health and lifestyle indicators (Spearman’s rho).

Variables	Spearman’s ρ	*p*-Value
Q-VAS ↔ WHOQOL-BREF Physical	0.55	<0.01
EQ-VAS ↔ WHOQOL-BREF Psychological	0.48	<0.01
BMI ↔ WHOQOL-BREF Physical	−0.42	<0.05

Note: Non-parametric correlations based on Spearman’s rank coefficient. Given the exploratory design and the limited sample size (N = 40), these findings should be considered preliminary and primarily intended for hypothesis generation.

## Data Availability

The datasets generated and analyzed during the current study are available from the corresponding author upon reasonable request.

## Abbreviations

The following abbreviations are used in this manuscript:

QoL	Quality of Life
HRQoL	Health-Related Quality of Life
LMIC	Low- and Middle-Income Countries
SD	Standard Deviation
